# Oxygen Enrichment Ameliorates Cardiorespiratory Alterations Induced by Chronic High-Altitude Hypoxia in Rats

**DOI:** 10.3389/fphys.2020.616145

**Published:** 2021-01-07

**Authors:** Xi Shao, Xu Dong, Jing Cai, Chi Tang, Kangning Xie, Zedong Yan, Erping Luo, Da Jing

**Affiliations:** ^1^ Department of Biomedical Engineering, Fourth Military Medical University, Xi’an, China; ^2^ Recuperation Management Office, Department of Medical Management and Training, Qingdao Special Service Recuperation Center of PLA Navy, Qingdao, China; ^3^ College of Basic Medicine, Shaanxi University of Chinese Medicine, Xianyang, China

**Keywords:** oxygen enrichment, cardiorespiratory system, high-altitude hypoxia, chronic mountain sickness, pulmonary hypertension

## Abstract

Chronic high-altitude hypoxia (HAH) results in compensatory pathological adaptations, especially in the cardiorespiratory system. The oxygen enrichment technology can provide long-lasting oxygen supply and minimize oxygen toxicity, which has proven to be effective to increase oxygen saturation, decrease heart rate, and improve human exercise performance after ascending to high altitudes. Nevertheless, it remains unknown whether oxygen enrichment can resist chronic HAH-induced cardiorespiratory alterations. Thirty-six male rats were equally assigned to the normal control (NC), HAH, and HAH with oxygen enrichment (HAHO) groups. The HAH and HAHO rats were housed in a hypobaric hypoxia chamber equivalent to 5,000 m for 4 weeks. The HAHO rats were exposed to oxygen-enriched air for 8 h/day. We found that oxygen enrichment mitigated the augmented skin blood flow and improved the locomotor activity of HAH-exposed rats. Oxygen enrichment inhibited HAH-induced increase in the production of red blood cells (RBCs). The hemodynamic results showed that oxygen enrichment decreased right ventricular systolic pressure (RVSP) and mean pulmonary artery pressure (mPAP) in HAH-exposed rats. HAH-associated right ventricular hypertrophy and cardiomyocyte enlargement were ameliorated by oxygen enrichment. Oxygen enrichment inhibited HAH-induced excessive expression of cytokines associated with cardiac hypertrophy and myocardial fibrosis [angiotensin-converting enzyme (ACE)/angiotensin-converting enzyme 2 (ACE2), angiotensin II (Ang II), collagen type I alpha 1 (Col1α1), collagen type III alpha 1 (Col3α1), and hydroxyproline] in the right ventricle (RV). Oxygen enrichment inhibited medial thickening, stenosis and fibrosis of pulmonary arterioles, and cytokine expression related with fibrosis (Col1*α*1, Col3α1, and hydroxyproline) and pulmonary vasoconstriction [endothelin-1(ET-1)] in HAH-exposed rats. This study represents the first effort testing the efficacy of the oxygen enrichment technique on cardiopulmonary structure and function in chronic HAH animals, and we found oxygen enrichment has the capability of ameliorating chronic HAH-induced cardiopulmonary alterations.

## Introduction

High altitude is defined as an elevation greater than 2,500 m above sea level with decreased barometric pressure and air density (Hackett and Roach, 2001). Although the proportion of oxygen remains constant, inspired partial pressure of oxygen decreases progressively with the falling barometric pressure at high altitudes (West, 2012). The reduced ambient oxygen pressure induces decrease in the arterial partial pressure of oxygen and arterial oxygen saturation, leading to subsequent oxygen deprivation of tissues, which is known as high-altitude hypoxia (HAH; Murray et al., 2018). Over 140 million people live at high-altitude regions exceeding 2,500 m, and numerous lowlanders commute to high altitudes for work or entertainment (Moore, 2001; West, 2015). Migrants ascending to high altitudes often suffer from acute mountain sickness with symptoms, like headache, insomnia, and fatigue (Hackett and Roach, 2001), while native or longtime dwellers at high altitudes have a high risk of developing chronic mountain sickness due to compensatory adaptation of organism to chronic HAH, and the risk is greater as the altitude increases (Azad et al., 2017). Exaggerated polycythemia represent the key characteristic of acclimation to chronic HAH, which is associated with increased blood viscosity and blood volume (León-Velarde and Richalet, 2006; Tremblay et al., 2019). Long-term HAH exposure also results in pulmonary hypertension with increased pulmonary vascular resistance due to pulmonary vasoconstriction and vascular remodeling (Penaloza and Arias-Stella, 2007). Pulmonary hypertension increases right ventricular afterload, and thus results in compensatory cardiac structural changes, characterized by right ventricular hypertrophy (Vonk Noordegraaf et al., 2017). Individuals exposed to chronic HAH also suffer from deteriorated cardiac function, such as increased heart rate, decreased stroke volume, and maximal cardiac output (Boussuges et al., 2000; León-Velarde et al., 2010). Chronic HAH-induced cardiorespiratory abnormalities are usually irreversible, which greatly reduces the quality of life and shortens life expectancy of highlanders (Vargas and Spielvogel, 2006; Yang et al., 2015). Therefore, it is of great significance to develop effective prevention strategies to protect cardiopulmonary function and reduce the risk of chronic HAH-associated cardiorespiratory diseases for native and long-term residents at high altitudes.

Several techniques have been reported to mitigate the detrimental effects induced by HAH, such as hyperbaric oxygen chamber, portable hyperbaric chamber, bottled oxygen, and oxygen concentrator based on molecular sieve. It has been demonstrated that hyperbaric oxygen chamber has the capability of increasing arterial oxygen saturation and attenuating HAH-related sickness (Kasic et al., 1991; Zhu et al., 2015). Nonetheless, the inflexibility and potential risks (e.g., middle ear barotrauma, temporary myopia, and pulmonary dyspnea) restrict its widespread application at high altitudes (Heyboer et al., 2017). The portable hyperbaric chamber has been proven effective to improve the symptoms of HAH, whereas the long-duration stays in the chamber may lead to issues like vomiting and claustrophobia (Bärtsch, 1992; Davis and Hackett, 2017). Although bottled oxygen (oxygen gas or liquid oxygen) can offer high-concentration oxygen to ameliorate HAH-induced adverse effects (Peacock, 1998), this approach has some negligible limitations, such as the potential oxygen poisoning and inability of long-run supply (Deneke and Fanburg, 1980). Oxygen concentrator based on molecular sieve can remove the nitrogen from air to obtain high-concentration oxygen (80–95%), which has been widely used for long-term oxygen therapy (Bolton et al., 2006; Nasilowski et al., 2008). However, the low flow rate and potential oxygen toxicity may be its major limitations (Adde et al., 2013). A growing body of studies has raised the interest in the technology of oxygen enrichment, which has proven to be an effective strategy to decrease the equivalent altitude (every 1% increase in oxygen concentration results in the reduction in equivalent altitude by about 300 m; West, 2002, 2003, 2012). This technique can not only provide long-lasting supply of oxygen enriched air with high flow rate, but also minimize the potential oxidative stress injury (Nagatomo et al., 2012). Recently, our group has developed a series of new oxygen enrichment instruments (including the manpack version, vehicle-mounted version, and room-mounted version) based on the membrane gas separation technique (Shen et al., 2013). Studies from our and other groups have shown that the oxygen enrichment technique has the capability of improving the oxygen saturation and decreasing the heart rate of individuals exposed to HAH (Mcelroy et al., 2000; Shen et al., 2013; Moraga et al., 2018). However, little is known about the efficacy of oxygen enrichment for chronic mountain sickness, especially for chronic HAH-induced damage to the cardiopulmonary system either experimentally or clinically. Therefore, it is necessary to identify the therapeutic effects of the oxygen enrichment technique on chronic HAH-induced cardiorespiratory dysfunction in animals, which can provide rationale for future clinical trials as well as reasonable and scientific application for individuals at high altitudes.

In this study, the chronic HAH rat model was established in a hypobaric hypoxia chamber and then exposed to oxygen-enriched air using on our oxygen enrichment device. Then, the effects of oxygen enrichment on the cardiovascular and pulmonary systems in chronic HAH rats were evaluated *via* systemic behavioral, hematological, histomorphological, and molecular assessments. The present study represents the first effort to characterize the potential effects of oxygen enrichment on chronic HAH-induced alterations in cardiorespiratory structure and function in animals.

## Materials and Methods

### Animals and Experimental Design

**Figure 1:**
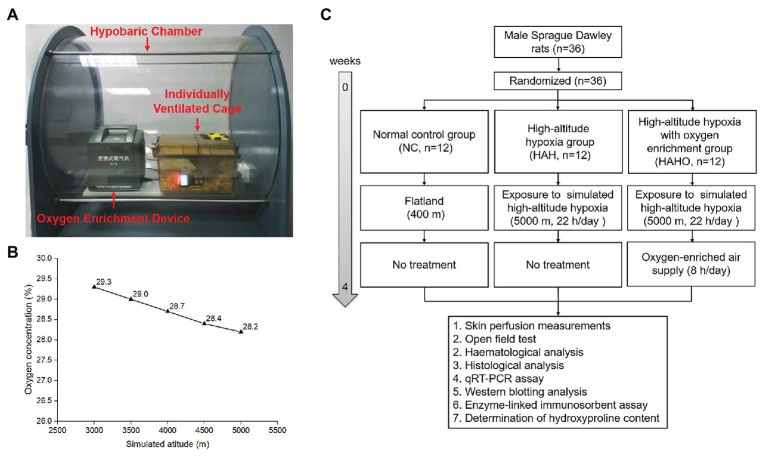
Experimental setup and experimental protocol employed in the present study. **(A)** Local oxygen-enriched environment was constructed in the hypobaric hypoxia chamber with the combination of the portable oxygen enrichment device and individually ventilated cages (IVCs) for rats. **(B)** The oxygen concentration in the gas generated by the oxygen enrichment device at various simulated altitudes (3,000–5,000 m). **(C)** The flow diagram of the experimental protocol used in this study.

Thirty-six male Sprague-Dawley rats (7–8 weeks old, weighing 260–320 g) used in the present study were obtained from the Animal Center of the Fourth Military Medical University. Rats were housed under controlled temperature (23 ± 1°C) and humidity (40–60%) with a 12 h light/dark cycle. Animal experiments were performed in accordance with the Guide for the Care and Use of Laboratory Animals published by the National Institutes of Health. Animals were randomly assigned into the following groups: the normal control group (NC, *n* = 12), the HAH group (*n* = 12), and the HAH with oxygen enrichment group (HAHO, *n* = 12). Rats in the NC group were housed under normoxic conditions at an altitude of 400 m in the city of Xi’an. Rats in the HAH group and HAHO group were housed in the hypobaric hypoxia chamber with 22 h/day for 4 consecutive weeks, while rats in the HAHO group were subjected to oxygen enrichment treatment (8 h/day) using the oxygen enrichment device developed by our group for 4 weeks. The hypobaric chamber was kept open for 2 h per day to replenish food and water, change bedding, clean the chamber and cage, and set up the hypobaric chamber (Titus et al., 2007). The body weight, food intake, and water intake of rats in each group were measured weekly during the experiment. The overall experimental protocol of this study is illustrated in Figure 1.

### Hypobaric Hypoxia Exposure and Oxygen Enrichment

A commercial hypobaric hypoxia chamber (#LAT-SY01, Suzhou Liante Medical Equipment Co., Ltd., Suzhou, China) was used with the chamber pressure maintained at 54 kPa and oxygen concentration at 20.9% to simulate the barometric pressure equivalent to an altitude of 5,000 m. A vacuum pump was used to evacuate the air out of the chamber. Solenoid valves were used to precisely control the proportion of gas into or out of the chamber to ensure adequate air circulation. The ascent and descent rates of the hypobaric hypoxia chamber were 91.3 Pa/s. Local oxygen-enriched environment was established in the hypobaric hypoxia chamber with the combination of a portable oxygen enrichment device and individually ventilated cages (IVC) for rats (Figure 1). The novel oxygen enrichment device was developed by our group based on the oxygen enrichment membrane technique (China Patent No. ZL201210309573.3 and ZL201310468909.5), which has been described in detail in our previous study (Shen et al., 2013). In brief, the device consisted of a ventilation fan, a vacuum pump, a gas buffer, and 16 parallelized oxygen enrichment membrane units. The surface of the oxygen enrichment membrane was coated with a selective layer of polymer solution for high oxygen permeance. Since oxygen permeates through the membrane faster than nitrogen, the membrane exhibits high oxygen/nitrogen selectivity. The vacuum controller was utilized to monitor the pressure of the oxygen-enriched air and suck the air out from membranes. The equipment can produce oxygen-enriched gas with steady oxygen concentration and flow rate both in the hypoxia chamber and in Tibet of China (Shen et al., 2013). The oxygen concentration of the enriched air was measured using an oxygen analyzer (OXYMAT 61, Siemens, Erlangen, Germany). According to the measurements, the oxygen concentration of the air generated by the device was maintained in the range of 28.2–29.3% at the simulated altitude from 3,000 to 5,000 m (Figure 1).

### Skin Perfusion Measurements

A Laser Doppler flowmetry (LDF, Moor Instruments Ltd., Axminster, United Kingdom) was utilized to measure the skin microvascular blood flux for all rats in each group following the manufacturer’s guidelines. Rats were anesthetized intraperitoneally using sodium pentobarbital (30 mg/kg body weight). After calibration, the laser probe (MP7-V2) was positioned on the plantar surface of the left hind paws with a laser wavelength of 780 nm. Rats were placed in the prone position and the probe was held for a period of 1 min. The cutoff frequency of the low-pass filter was set at 22.5 kHz and the time constant was defined as 0.1 s to minimize the interference. Data were recorded continuously at the sampling rate of 40 Hz and transferred to the laptop for further analysis based on the software provided by the manufacturer (moorVMS-PC software). Mean skin blood flux was measured and expressed in arbitrary units (AUs).

### Open Field Testing

Spontaneous locomotor activities of all rats in each group were assessed using the activity cage apparatus (Model 7,420, Ugo Basile, Verase, Italy), which comprised an electronic unit and two sets of emitter/sensor arrays to transmit and detect the invisible infrared beams. Rats were placed in the center of the cage and allowed to move freely. After the rats were acclimated to the apparatus for 10 min, the infrared beam interruptions caused by the movements of rats were automatically collected by the electronic unit for a period of 10 min. Data were analyzed for the measurements of the horizontal activity (crossed beams) and vertical activity (crossed beams).

### Hemodynamic Measurements

Hemodynamics of all rats in each group were measured by the right heart catheterization (RHC). After anesthesia with sodium pentobarbital, rats were stabilized on the operating table. An incision was created on the skin from the mandible to sternum. After surrounding tissues were gently dissected, the right external jugular vein was isolated and ligated proximally with a loose knot. Then, a small hole was created distally to the ligature. A catheter was inserted into the hole of the vein, and then the knot was tightened. The catheter, connected to a hemodynamic monitor (Hewlett-Packard, Palo Alto, CA, United States), was gently moved into the right ventricle (RV) and pulmonary artery to measure the right ventricular systolic pressure (RVSP, mmHg) and mean pulmonary artery pressure (mPAP, mmHg), respectively.

### Hematological Analysis

After the RHC measurement, cardiac puncture of all rats in each group was performed to collect blood from left ventricle (LV) using a 5 ml sterile syringe. Blood samples were transferred into sample bottles containing ethylenediaminetetraacetate (EDTA) and immediately analyzed by an automatic hematology analyzer (XN-3000, Sysmex, Kobe, Japan). Hematological parameters were determined as follows: red blood cell (RBC, 10^12^/L), hemoglobin (Hb, g/L), hematocrit (Hct, %), mean corpuscular volume (MCV, fl), mean corpuscular hemoglobin (MCH, pg), and white blood cell (WBC, 10^9^/L).

### Determination of Ventricular Weight

After cardiac puncture, heart tissues of all rats in each group were isolated and washed repeatedly with ice-cold phosphate-buffered saline (PBS) to remove the remaining blood. After removal of atria, pulmonary trunk, and vessels from the ventricles, the RV was isolated from the LV and ventricular septum. After completely drying PBS with filter papers, weights of the RV free wall, LV free wall, and ventricular septum were determined by an electronic balance (Tianchen instrument, Beijing, China). The heart weight (HW), left ventricular weight (LVW), and right ventricular weight (RVW) were measured. The RV weight to LV plus septum weight ratio [RV/(LV + S); Fulton index) was calculated as an index of RV hypertrophy.

### Histological Analysis

After sacrifice, cardiac muscle tissues from six rats in each group were used for histological analysis, and cardiac muscle tissues of the remaining six rats in each group were used for molecular expression assays. The right lung tissues from all rats in each group (*n* = 12) were used for histological analysis, and the left lung tissues were used for molecular expression assays. The isolated heart and right upper lobe tissues were fixed in 10% formalin overnight. Then, specimens were dehydrated with a graded series of ethanol, embedded in paraffin, and cut into 5-μm slices using a diamond saw microtome (Leica 2500E, Leica SpA, Milan, Italy). Specimens of the cardiac muscle tissues were stained with H&E and imaged under an optical microscope (Olympus, Tokyo, Japan) at 400× magnification to assess the myocardial morphology. The cross-sectional area (μm^2^) of the cardiomyocyte was determined using ImageJ (National Institutes of Health, Bethesda, Maryland, United States). For lung tissues, sections were stained with H&E and Van Gieson (VG) to observe the morphology and collagen deposition of small pulmonary arteries, respectively. Small pulmonary arteries (50–100 μm in diameter) were randomly selected to measure internal diameter (ID), external diameter (ED), vascular lumen area (VA), and total vascular area (TA). Then, the percentage of medial wall thickness (MWT%) was calculated as [MWT% = (ED−ID)/ED × 100%]. The percentage of vascular wall area (WA%) was calculated as [WA% = (TA−VA)/TA × 100%].

### Real-Time PCR

Total RNA was extracted from heart or lung tissues using TRIzol (Invitrogen, Carlsbad, CA, United States). The concentration and purity of RNA were confirmed by the UV spectrophotometer (Bio-Rad, Hercules, CA, United States). Total RNA was synthesized for cDNA using the PrimeScript™ RT Master Mix (Takara, Ohtsu, Japan) according to the manufacturer’s guidelines. The primers of hypoxia-inducible factor-1 alpha (HIF-1*α*), angiotensin-converting enzyme (ACE), angiotensin-converting enzyme 2 (ACE2), collagen type I alpha 1 (Col1α1), collagen type III alpha 1 (Col3α1), and GAPDH were designed using Primer Premier 5.0 (PREMIER Biosoft International, Palo Alto, CA, United States) and synthesized by Takara Biotechnology Co. Ltd. (Table 1). The RCR mix consisted of 10 μl SYBR Premix Ex Taq™ II (Takara), 1.6 μl cDNA, 1.6 μl of the upstream and downstream primers, and 6.8 μl dH_2_O in a final reaction volume of 20 μl. All PCR reactions were performed in the CFX96 Real-Time PCR Detection System (Bio-Rad) under the following conditions: initial denaturation at 95°C for 3 min, and followed by 40 cycles consisting of denaturation at 95°C for 30 s, annealing at 60°C for 30 s, and extension at 65°C for 10 s. The target gene expression was normalized to GAPDH using the 2^-*Δ*ΔCt^ method.

**Table 1:** The sequence of primers used in the present study for real-time PCR.

Genes	Primers	Primer sequence (5'-3')
HIF-1α	Forward	CCAGATTCAAGATCAGCCAGCA
	Reverse	GCTGTCCACATCAAAGCAGTACTCA
ACE	Forward	TGCCTCAGCCTGGGACTTCTA
	Reverse	CCCATTTCGTGGTGGGCTA
ACE2	Forward	GGTCTTCTGCCATCCAATTTTC
	Reverse	ACCATCCACCTCCACTTCTCTAAC
Col1α1	Forward	GACATGTTCAGCTTTGTGGACCTC
	Reverse	AGGGACCCTTAGGCCATTGTGTA
Col3α1	Forward	TTTGGCACAGCAGTCCAATGTA
	Reverse	GACAGATCCCGAGTCGCAGA
GAPDH	Forward	GGCACAGTCAAGGCTGAGAATG
	Reverse	ATGGTGGTGAAGACGCCAGTA

### Western Blotting

Total protein was extracted from cardiac muscle tissues using RIPA buffer supplemented with PMSF. The protein concentration was quantified using a BCA protein assay kit (DingguoChangsheng Biotech Co., Beijing, China). The protein was separated in 6% or 10% SDS-PAGE and transferred to the NC membrane (Millipore Corp., Bedford, Mass, United States) by electroblotting. Then, membranes were blocked in 5% skim milk for 1 h at room temperature and incubated at 4°C overnight with the primary antibodies of HIF-1*α*, ACE, ACE2, and GAPDH (Bioworld, Atlanta, GA, United States). After washing with Tris-buffered saline containing 0.5% Tween-20 (TBST), membranes were incubated with the secondary antibody for 2 h at room temperature. The blots were developed using the SuperSignal West Pico chemiluminescent substrate kit (Thermo Scientific, Rockford, IL, United States) and visualized by an ECL chemiluminescent system (GE ImageQuant 350, GE Healthcare).

### Enzyme-Linked Immunosorbent Assay

The protein levels of angiotensin II (Ang II) in myocardial tissues and endothelin-1 (ET-1) in lung tissues were evaluated using the ELISA kits (Nanjing Jiancheng Bioengineering Institute, Nanjing, China). Samples (100 mg) were homogenized in 900 μl of 1 × PBS (pH: 7.0–7.4) at 4°C. The homogenates were centrifuged at 12,000 r/min for 10 min, and the supernatant was collected for the measurements. All procedures of the ELISA assays were strictly performed according to the manufacturer’s protocol.

### Determination of Hydroxyproline Content

Samples from myocardial and lung tissues were evaluated for the quantification of hydroxyproline (Hyp) based on the alkaline hydrolysis method. After weighing, samples with 10 mg were detected using the hydroxyproline kit (Nanjing Jiancheng Bioengineering Institute, Nanjing, China). The absorbance of samples was measured spectrophotometrically at wavelength of 550 nm following the manufacturer’s guidelines. The Hyp (μg/mg wet weight) concentration was determined from the standard curve.

### Statistical Analysis

The statistical analysis was performed using the SPSS 19.0 software (SPSS, Chicago, IL, United States). Results were expressed as mean ± SD. Value of *p* < 0.05 was considered statistically significant. Normal distribution was assessed using the Kolmogorov-Smirnov test, and homoscedasticity was examined using the Levene’s test. All data were found to obey normal distribution and homoscedasticity. Because of the existed difference of body weights among the three groups, data of the HW, LVW, and RVW were compared using analysis of covariance (ANCOVA) with the body mass as a covariate. In additions to the heart and ventricular weights, comparisons of all the other data between each two groups were performed using one-way ANOVA followed by Bonferroni’s *post hoc* analysis. A power analysis was performed to obtain the minimum sample number to ensure the statistical significance among the three groups using the G*Power software. Using a one-way ANOVA with *α* = 0.05 and power = 0.8, we found that the least animal number in each group to establish significance was 4. Thus, six rats per group for the cardiac muscle histological analysis and myocardial cytokine expression analysis and 12 rats per group for the other tests in the present study were sufficient to secure a good statistical significance.

## Results

### Body Mass, Food and Water Intake, Skin Perfusion, and Locomotor Activity

**Figure 2:**
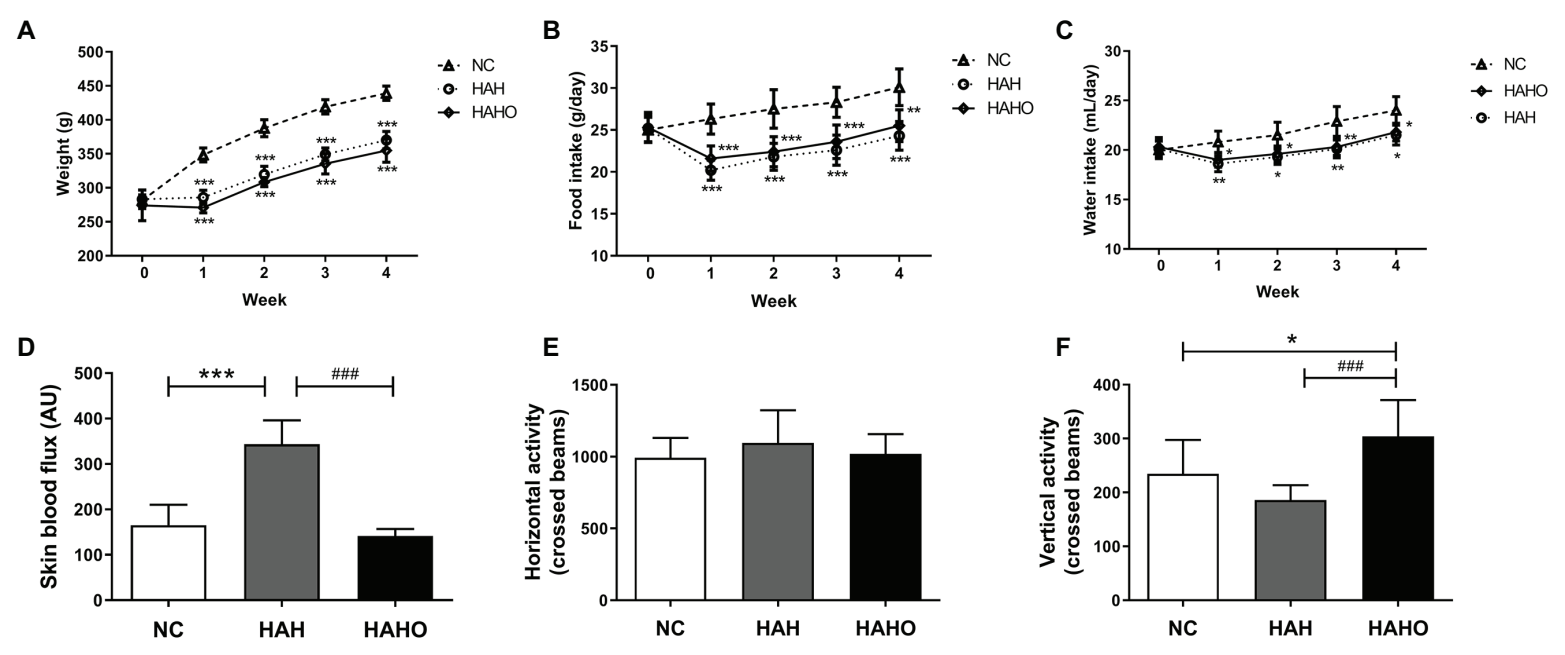
Effects of local oxygen enrichment on **(A)** body weight, **(B)** food intake, **(C)** water intake, **(D)** skin perfusion, and **(E,F)** locomotor activity of rats exposed to 4-week hypobaric hypoxia. All data are expressed as mean ± SD (*n* = 12). NC, normal control group; HAH, high-altitude hypoxia group; and HAHO, HAH with oxygen enrichment group. ^*^p < 0.05 vs. the NC group, ^**^p < 0.01 vs. the NC group, ^***^p < 0.001 vs. the NC group, and ^###^*p* < 0.001 vs. the HAH group.

As shown in Figures 2A–C, no significant difference in body weights, food intake, and water intake was observed before the experiment (*p* > 0.05). Body weights, food intake, and water intake of rats in the HAH and HAHO groups were significantly lower than those in the NC group at Weeks 1–4 post hypobaric hypoxia exposure (*p* < 0.05). However, body weights, food intake, and water intake between the HAH group and HAHO group showed no significant difference at any time point (*p* > 0.05). The HAH group exhibited significant increase in the skin blood flux as compared with the NC group (Figure 2D, *p* < 0.001), while the HAHO group showed significantly lower skin blood flux than the HAH group (*p* < 0.001). No significant difference was found in the horizontal activity among the three groups (Figure 2E, *p* > 0.05). Moreover, no statistical significance was observed in the vertical activity of rats between the HAH group and NC group (Figure 2F, *p* > 0.05). However, the HAHO group showed significant increase in the vertical activity as compared with the NC group and the HAH group (*p* < 0.05).

### Hematological Analysis

**Figure 3:**
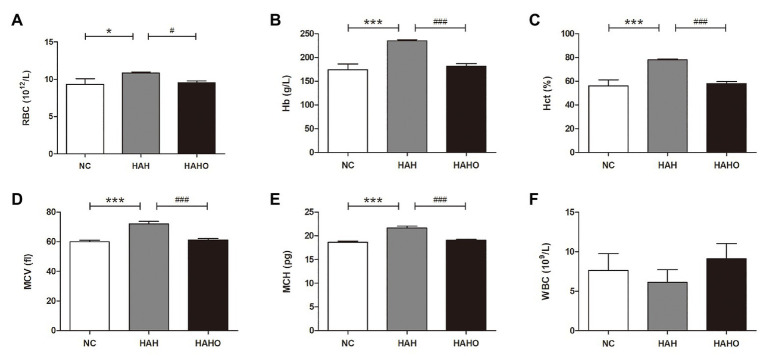
Effects of local oxygen enrichment on hematological parameters of rats exposed to 4-week hypobaric hypoxia, including **(A)** red blood cell (RBC), **(B)** hemoglobin (Hb), **(C)** hematocrit (Hct), **(D)** mean corpuscular volume (MCV), **(E)** mean corpuscular hemoglobin (MCH), and **(F)** white blood cell (WBC). All data are expressed as mean ± SD (*n* = 12). NC, normal control group; HAH, high-altitude hypoxia group; and HAHO, high-altitude hypoxia with oxygen enrichment group. ^*^p < 0.05 *vs.* the NC group, ^***^p < 0.001 vs. the NC group, ^#^*p* < 0.05 vs. the HAH group, and ^###^*p* < 0.001 vs. the HAH group.

According to the hematological results of RBC indices, the HAH group had significantly higher RBC (*p* < 0.05), Hb (*p* < 0.001), Hct (*p* < 0.001), MCV (*p* < 0.001), and MCH (*p* < 0.001) than the NC group (Figures 3A–E). Oxygen enrichment resulted in remarkable decrease in RBC (*p* < 0.05), Hb (*p* < 0.001), Hct (*p* < 0.001), MCV (*p* < 0.001), and MCH (*p* < 0.001) in HAH-exposed rats. Furthermore, no significant change in these erythrocyte indices (RBC, Hb, Hct, MCV, and MCH) was found between the HAHO group and the NC group (*p* > 0.05). Moreover, no significant difference was observed in WBC among the three groups (Figure 3F, *p* > 0.05).

### Hemodynamic and Ventricular Weight Assessment

**Figure 4:**
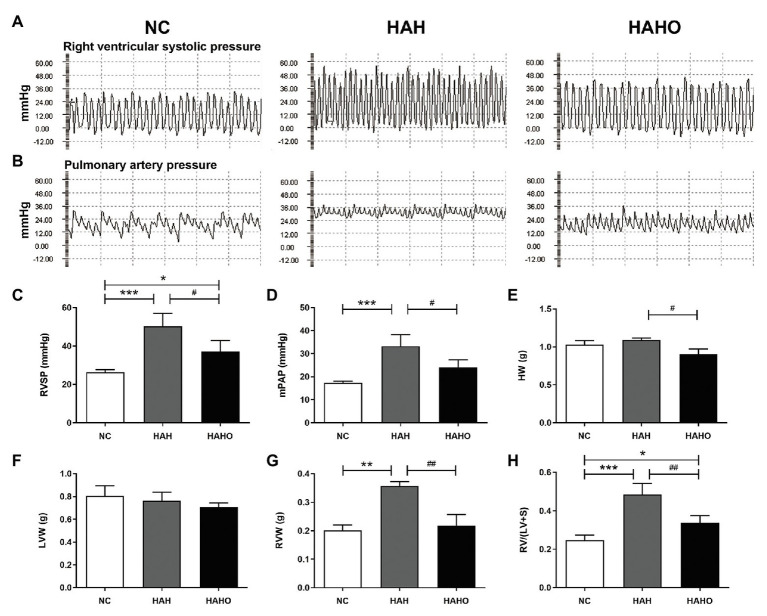
Effects of local oxygen enrichment on hemodynamic indices and ventricular weight of rats exposed to 4-week hypobaric hypoxia. **(A,B)** Representative images of right ventricular systolic pressure (RVSP) and mean pulmonary artery pressure (mPAP). **(C,D)** Statistical results of hemodynamic parameters, including RVSP and mPAP. **(E–H)** Statistical results of ventricular weight, including the heart weight (HW), left ventricular weight (LVW), right ventricular weight (RVW), and the RV weight to LV plus septum weight ratio [RV/(LV + S)]. All data are expressed as mean ± SD (*n* = 12). NC, normal control group; HAH, high-altitude hypoxia group; and HAHO, high-altitude hypoxia with oxygen enrichment group. ^*^p < 0.05 vs. the NC group, ^**^p < 0.05 vs. the NC group, ^***^p < 0.001 vs. the NC group, ^#^*p* < 0.05 vs. the HAH group, and ^##^*p* < 0.01 vs. the HAH group.

The representative images of hemodynamic results (Figures 4A,B) demonstrated that the levels of RVSP and mPAP in the HAH group were significantly increased as compared with the NC group (Figures 4C,D, *p* < 0.001). The RVSP and mPAP levels of rats in the HAHO group were significantly lower than those in the HAH group (*p* < 0.05). The HAHO group showed significantly higher RVSP than the NC group (*p* < 0.05). The HAH group exhibited significantly higher RVW (*p* < 0.01) and RV/(LV + S; *p* < 0.001) than the NC group (Figures 4G,H), indicating that RV hypertrophy occurred in rats exposed to hypoxia. In comparison with the HAH group, the HAHO group showed significantly lower HW (*p* < 0.05), RVW (*p* < 0.01), and RV/(LV + S; *p* < 0.01). The HAHO group showed significant increase in RV/(LV + S; *p* < 0.05), whereas no significant difference was found in HW or RVW between the HAHO group and the NC group (Figures 4E–H, *p* > 0.05). No significant difference in LVW was detected among the three groups (Figure 4F, *p* > 0.05).

### Cardiac Muscle Histology

**Figure 5:**
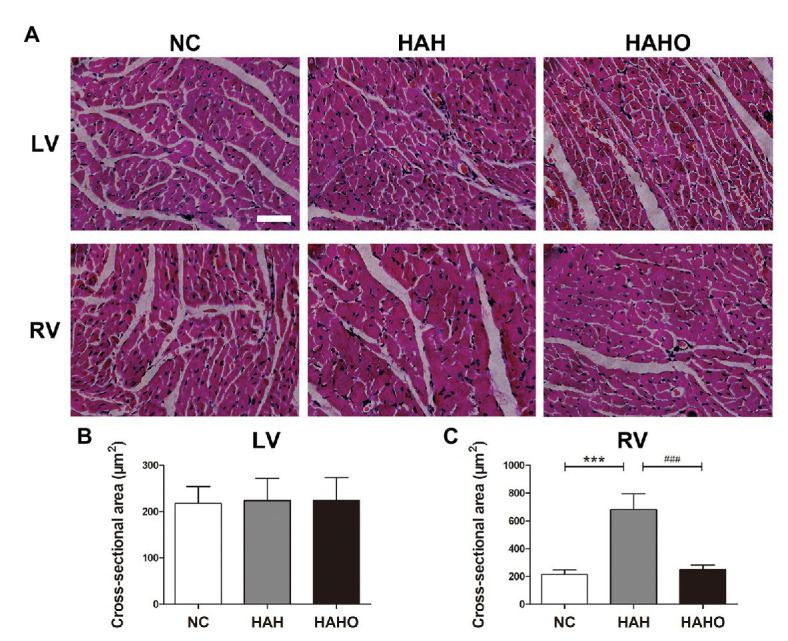
Effects of local oxygen enrichment on the cardiomyocytes of rats exposed to 4-week hypobaric hypoxia according to the histological analysis. **(A)** H&E staining images of the cross-sections of the left ventricle (LV) and right ventricle (RV). Scale bars = 50 μm. **(B,C)** Statistical results of the cross-sectional area for the LV and RV cardiomyocytes. All data are expressed as mean ± SD (*n* = 6). NC, normal control group; HAH, high-altitude hypoxia group; and HAHO, high-altitude hypoxia with oxygen enrichment group. ^***^p < 0.001 vs. the NC group, ^###^*p* < 0.001 vs. the HAH group.

The H&E staining of cardiac muscle (Figure 5A) demonstrated that 4-week hypobaric hypoxia exerted no significant effect on the LV, as evidenced by no statistically significant difference in the cross-sectional area of LV cardiomyocytes between the NC group and the HAH group (Figure 5B, *p* > 0.05). However, the cross section of the RV myocardium showed observable cardiomyocyte enlargement in the HAH group as compared with the NC group (Figure 5A), which was confirmed by the significant increase in the RV cardiomyocyte cross-sectional area (Figure 5C, *p* < 0.001). No significant difference was found in the cross-sectional area of cardiomyocytes in the LV between the HAHO group and the HAH group (*p* > 0.05). The HAHO group showed significant decrease in the cross-sectional area of RV cardiomyocytes as compared with the HAH group (*p* < 0.001). In addition, no significant change was observed in the cross-sectional area of RV cardiomyocytes between the HAHO group and the NC group (*p* > 0.05).

### Myocardial Cytokine Expression

**Figure 6:**
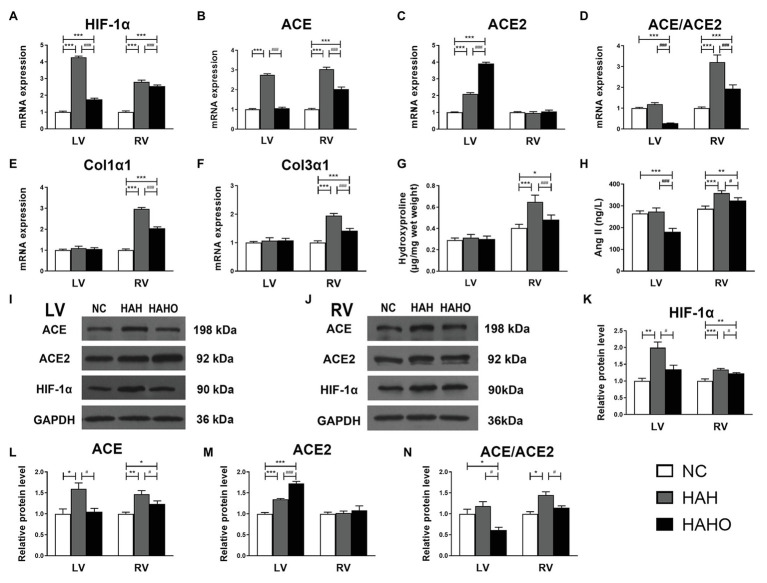
Effects of local oxygen enrichment on the gene expression and protein production of critical cytokines in the LV and RV of rats exposed to 4-week hypobaric hypoxia. **(A–F)** Statistical results for the mRNA expression of HIF-1*α*, ACE, ACE2, ACE/ACE2, Col1α1, and Col3α1 based on the real-time PCR assays. **(G)** Quantification of myocardial hydroxyproline concentration based on the alkaline hydrolysis method. **(H)** Evaluation of myocardial angiotensin II (Ang II) based on ELISA. **(I,J)** Representative western blotting images for the protein expression of HIF-1α, ACE, ACE2, and GAPDH in the LV and RV. **(K–N)** Statistical results for the relative protein levels of HIF-1α, ACE, ACE2, and ACE/ACE2 in the LV and RV. All data are expressed as mean ± SD (*n* = 6). NC, normal control group; HAH, high-altitude hypoxia group; and HAHO, high-altitude hypoxia with oxygen enrichment group. ^*^p < 0.05 vs. the NC group, ^**^p < 0.01 vs. the NC group, ^***^p < 0.001 vs. the NC group, ^#^*p* < 0.05 vs. the HAH group, and ^###^*p* < 0.001 vs. the HAH group.

The HAH group exhibited significantly higher messenger RNA (mRNA) levels of HIF-1*α*, ACE, and ACE2 in the LV than the NC group (Figures 6A–C, *p* < 0.001). However, no significant difference was observed in the ACE/ACE2 mRNA ratio between the HAH group and the NC group (Figure 6D, *p* > 0.05). The HAHO group had significantly lower mRNA expression of HIF-1α, ACE, and ACE/ACE2 ratio (*p* < 0.001) and higher ACE2 expression (*p* < 0.001) in the LV than the HAH group. In comparison with the NC group, the HAHO group showed significantly increased gene expression of HIF-1α and ACE2, and decreased ACE/ACE2 mRNA ratio in the LV (*p* < 0.001). Nevertheless, no significant difference was observed in Col1α1 or Col3α1 gene expression in the LV among the three groups (Figures 6E,F, *p* > 0.05). The HAH group showed significantly higher mRNA levels of HIF-1*α*, ACE, ACE/ACE2 ratio, Col1α1, and Col3α1 in the RV than the NC group (Figures 6A–F, *p* < 0.001). The HAHO group exhibited significant decrease in HIF-1α, ACE, ACE/ACE2 ratio, Col1α1, and Col3α1 gene expression in the RV as compared with the HAH group (Figures 6A–F, *p* < 0.001). The mRNA levels of HIF-1*α*, ACE, ACE/ACE2 ratio, Col1α1, and Col3α1 in the RV of the HAHO group were significantly higher than those in the NC group (*p* < 0.001). However, no difference was found in the ACE2 gene expression in the RV among the three groups (Figure 6C, *p* > 0.05).

Based on the alkaline hydrolysis method, no significant difference was observed in the hydroxyproline content in the LV among the three groups (Figure 6G, *p* > 0.05). However, the hydroxyproline concentration in the RV of the HAH group was significantly higher than that in the NC group (*p* < 0.001). Oxygen enrichment significantly decreased the hydroxyproline content in the RV of the HAHO group as compared with the HAH group (*p* < 0.001). The HAHO group exhibited significantly higher hydroxyproline content than that in the NC group (*p* < 0.05). The ELISA results showed no significant change in the cardiac Ang II level in the LV (Figure 6H, *p* > 0.05) between the NC group and the HAH group, whereas the HAH group showed a significant increase in myocardial Ang II concentration in the RV as compared with the NC group (*p* < 0.001). The HAHO group exhibited significantly lower Ang II in the LV (*p* < 0.001) and RV (*p* < 0.05) as compared with the HAH group. The HAHO group showed significant decrease in cardiac Ang II concentration in the LV (*p* < 0.001), and significantly increased Ang II level in the RV (*p* < 0.01).

The Western blotting results (Figures 6I,J) suggested that the protein levels of HIF-1*α*, ACE, and ACE2 in the LV of the HAH group were significantly higher than those in the NC group (Figures 6K–M, *p* < 0.05). The HAH group exhibited no significant difference in the ACE/ACE2 protein ratio as compared with the NC group (Figure 6N, *p* > 0.05). The HAHO group showed significantly decreased HIF-1α, ACE, and ACE/ACE2 ratio (*p* < 0.05), and increased ACE2 protein expression (*p* < 0.001) in the LV as compared with the HAH group. The HAHO group exhibited significantly higher protein level of ACE2 (*p* < 0.001) and significantly lower ACE/ACE2 protein ratio (*p* < 0.05) than the NC group, whereas no significant difference was observed in the HIF-1α or ACE protein expression between the HAHO group and the NC group (*p* > 0.05). HAH resulted in a significant increase in the HIF-1*α* (*p* < 0.001) and ACE protein expression (*p* < 0.01) and significantly increased ACE/ACE2 protein ratio (*p* < 0.05), whereas HAH had no effect on the ACE2 protein expression in the RV (*p* > 0.05) of the HAH group as compared with the NC group. Although the HAHO group exhibited significant decrease in the protein levels of HIF-1*α*, ACE, and ACE/ACE2 ratio (*p* < 0.05), no significant difference in the ACE2 protein expression (*p* > 0.05) was observed in the RV as compared with the HAH group. The HAHO group showed significantly higher protein expression of HIF-1*α* (*p* < 0.01) and ACE (*p* < 0.05) than the NC group, whereas no significant difference was found in the protein expression of ACE2 or ACE/ACE2 between the HAHO group and the NC group.

### Lung Histology

**Figure 7:**
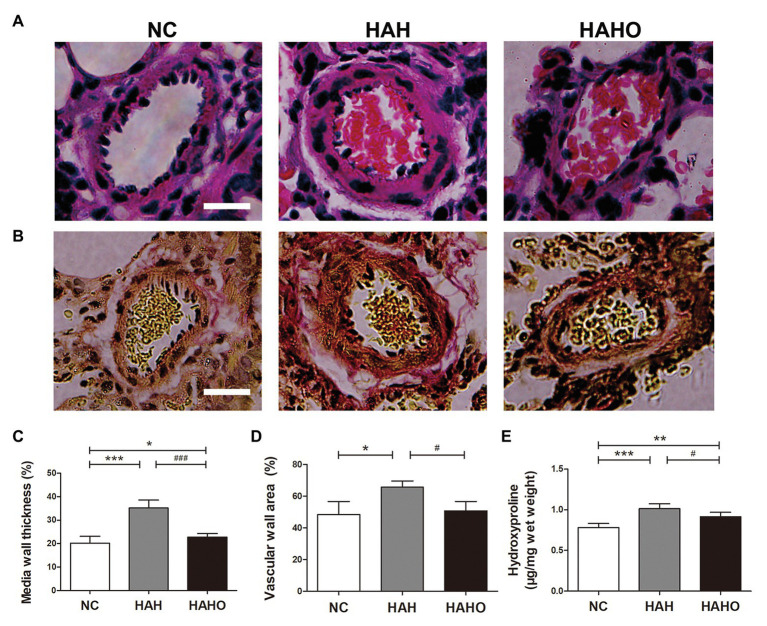
Effects of local oxygen enrichment on the pulmonary arterioles of rats exposed to 4-week hypobaric hypoxia. **(A,B)** Representative H&E and Van Gieson (VG) staining images of pulmonary arterioles. Scale bars = 20 μm. **(C,D)** Statistical results of the percentage of medial wall thickness (MWT%) and percentage of vascular wall area (WA%). MWT% was calculated as [external diameter (ED) − internal diameter (ID)]/external diameter × 100%. WA% was calculated as [total vascular area (TA) − vascular lumen area (VA)]/TA × 100%. **(E)** Quantification of hydroxyproline concentration in lung tissues based on the alkaline hydrolysis method. All data are expressed as mean ± SD (*n* = 12). NC, normal control group; HAH, high-altitude hypoxia group; and HAHO, high-altitude hypoxia with oxygen enrichment group. ^*^p < 0.05 vs. the NC group, ^**^p < 0.01 vs. the NC group, ^***^p < 0.001 vs. the NC group, ^#^*p* < 0.05 vs. the HAH group, and ^###^*p* < 0.001 vs. the HAH group.

According to the H&E staining images (Figure 7A), the HAH group exhibited increased medial wall thickness of pulmonary arterioles and stenosis as compared with the NC group. In addition, collagen deposition in the small pulmonary arteries was greater in the HAH group than the NC group (Figure 7B). Statistical analysis showed that the HAH group exhibited significantly increased MWT and WA as compared with the NC group (Figures 7C,D, *p* < 0.05). Moreover, the HAH group showed significantly higher hydroxyproline concentration in lung tissues than the NC group (Figure 7E, *p* < 0.001). These changes in the lung histology were partially prevented by oxygen enrichment. In comparison with the HAH group, the HAHO group demonstrated significant decrease in MWT (*p* < 0.001) and WA (*p* < 0.05). Although significant decrease in the hydroxyproline concentration was observed in the HAHO group as compared with the HAH group (*p* < 0.05), the HAHO group showed significantly higher hydroxyproline concentration in lung tissues than the NC group (*p* < 0.01).

### Intrapulmonary Cytokine Expression

**Figure 8:**
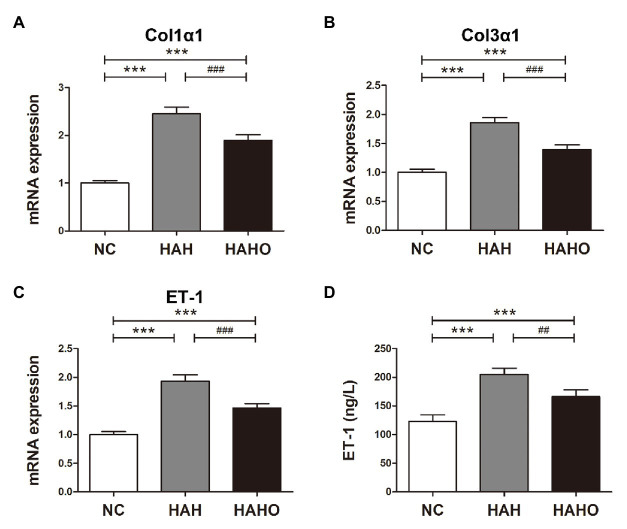
Effects of local oxygen enrichment on the gene expression and protein production of cytokines in lung tissues of rats exposed to 4-week hypobaric hypoxia. **(A–C)** Statistical results for the mRNA expression of Col1α1, Col3α1, and endothelin-1 (ET-1) based on the real-time PCR analysis. **(D)** Evaluation of the ET-1 protein production in lung tissues based on ELISA. All data are expressed as mean ± SD (*n* = 12). NC, normal control group; HAH, high-altitude hypoxia group; and HAHO, high-altitude hypoxia with oxygen enrichment group. ^***^p < 0.001 vs. the NC group, ^##^*p* < 0.01 vs. the HAH group, and ^###^*p* < 0.001 vs. the HAH group.

The lung mRNA levels of the Col1α1, Col3α1, and ET-1 in the HAH group were significantly higher than those in the NC group (Figures 8A–C, *p* < 0.001). Moreover, the HAH group showed significant increase in the ET-1 pulmonary concentration as compared with the NC group (Figure 8D, *p* < 0.001). Oxygen enrichment inhibited these negative changes in lung tissues, as evidenced by significantly decreased gene expression of Col1α1 (*p* < 0.001), Col3α1 (*p* < 0.001), and ET-1 (*p* < 0.001), and reduced ET-1 protein products in the HAHO group (*p* < 0.01) as compared with the HAH group. However, the mRNA levels of Col1α1, Col3α1, and ET-1, and the protein concentration of ET-1 in lung tissues were significantly higher than those in the NC group (*p* < 0.001).

## Discussion

The current available studies for the effects of oxygen enrichment on altitude sickness are mainly clinical investigations, in which suppressed heart rate and diastolic arterial pressure, and increased arterial oxygen saturation resulted from oxygen enrichment were observed for workers at high altitudes (Mcelroy et al., 2000; Li and Liu, 2014; Moraga et al., 2014, 2018). Our group has developed a novel oxygenation device based on the oxygen enrichment membrane technique, and we found that this novel device can induce higher saturation of peripheral oxygen, lower heart rate, and better physical work capacity (Shen et al., 2013). However, to our knowledge, no study has systematically investigated the effects of oxygen enrichment on the injuries of cardiorespiratory structure and function induced by chronic HAH. Based on systematic hematological, histomorphological, and molecular assessments, our results revealed that oxygen enrichment reversed the changes in erythrocyte parameters, largely preserved the cardiac structure and function, and significantly prevented the abnormalities in pulmonary arterioles (including pulmonary vascular remodeling and vasoconstriction) in rats exposed to chronic HAH.

Substantial evidence demonstrates that chronic HAH leads to the body weight reduction in humans or animals (Lippl et al., 2010). Similarly, we observed a significant reduction in the body weight, food, and water intake of rats exposed to chronic HAH, which may result from accelerated metabolic rate and impaired appetite (Tschöp and Morrison, 2001). However, oxygen enrichment for 8 h per day exerted no obvious effect on HAH-induced decrease in the body mass, food, and water consumption. The increased oxygen supply might not sufficiently compensate for the alteration of digestive metabolism at high altitudes. Moreover, we found that HAH augmented cutaneous microvascular blood flow, which was consistent with previous studies (Carey et al., 2019). Interestingly, oxygen enrichment reversed the increase in the skin blood flow caused by chronic HAH, probably because the increased blood oxygen level was sufficient to compensate for the oxygen consumption without producing microvascular adaptive changes under hypoxic conditions. The open field test has proven to be a useful technique to assess locomotion, exploration, and anxiety of animals (Walsh and Cummins, 1976; Carola et al., 2002; Tatem et al., 2014). Our results demonstrate that chronic HAH induced changes in neither horizontal nor vertical movements, whereas oxygen enrichment greatly increased the locomotor activity, revealing the potential beneficial effects of oxygen enrichment on behavioral responses in chronic HAH rats (Robinson et al., 2018).

It is well known that remarkable alterations occur in hematology to accommodate the HAH condition (Berglund, 1992; Huang et al., 2017). Chronic HAH-induced excessive erythrocytosis increases the blood viscosity, leading to the increased risk of various cardiovascular disorders (e.g., hypertension, vascular thrombosis, and myocardial infarction; Lowe et al., 1997; Tremblay et al., 2019). Our hematological indices revealed that chronic HAH for 4 weeks increased the amounts of overall RBCs and hemoglobin as well as the volume percentage of RBCs in the blood. Moreover, HAH also increased the average RBC size and the amount of hemoglobin per RBC, as evidenced by augmented MCV and MCH. However, HAH exerted no obvious effect on the number of WBCs. More importantly, our results demonstrated that oxygen enrichment significantly inhibited the increase in the production and volume of erythrocytes and expression of hemoglobin, and almost restored all these parameters to control levels. These findings reveal that oxygen enrichment is able to reverse the changes in erythrocyte parameters induced by chronic HAH, which is potentially beneficial for the decrease in the blood viscosity and subsequent decline in blood pressure (Letcher et al., 1981; Fowkes et al., 1993; Stauffer et al., 2020).

Vascular remodeling and vasoconstriction are common pathological changes under chronic HAH conditions, resulting in the development of high-altitude pulmonary hypertension (Sylvester et al., 2012; Wilkins et al., 2015). We found that chronic HAH induced thicker medial smooth muscles and severer stenosis of pulmonary arterioles, revealing pathological vascular remodeling in HAH rats. Our findings also showed that HAH greatly increased pulmonary mRNA expression and protein products of ET-1, which is a powerful vasoconstrictor involved in hypoxia-induced pulmonary vascular remodeling and pulmonary hypertension (Jankowich et al., 2016; Kylhammar and Rådegran, 2017). We also found that collagen fibers and expression levels of Col1*α*1, Col3α1, and hydroxyproline were significantly increased after chronic HAH, revealing the increased collagen synthesis and development of pulmonary fibrosis in lung tissues (Bernard, 2018; Li and Wu, 2018). Moreover, we observed that medial pulmonary thickness and expression of ET-1 in rat lung tissues were decreased after oxygen enrichment exposure, revealing that HAH-induced pulmonary vasoconstriction and pulmonary vascular remodeling were inhibited. Oxygen enrichment also mitigated HAH-mediated pulmonary fibrosis, as evidenced by decreased expression of Col1*α*1, Col3α1, and hydroxyproline in lung tissues. Previous studies have reported that oxygen enrichment remarkably improves the pulmonary function of subjects exposed to HAH, as evidenced by decreased breaths per minute and reduced incidence of sleep apneas (Luks et al., 1998; Li and Liu, 2014). Our current findings provide further evidence that oxygen enrichment is able to effectively resist chronic HAH-induced negative changes in pulmonary morphology and key molecular expression.

Pulmonary vasoconstriction and increased pulmonary vascular resistance induce adverse effects on the structure and function of RV under long-term HAH exposure (Wang and Chesler, 2013). Our hemodynamic measurements demonstrated that chronic HAH elevated pulmonary artery pressure and RV systolic pressure in rats, which was in line with previous studies (Naeije and Vanderpool, 2013; Boos et al., 2016; Dunham-Snary et al., 2017). It has been shown that the excessive hemodynamic burden imposed on RV leads to subsequent hypertrophic response (Van Der Bruggen et al., 2017; De Man et al., 2019). Our results demonstrated that 4-week HAH exposure remarkably increased the RV weight, Fulton index (an index for RV hypertrophy), and degree of RV cardiomyocyte hypertrophy in rats, whereas LV exhibited no similar changes after HAH exposure. Consistent with our results, HAH-associated RV dysfunction was also reported in previous clinical and experimental studies (Heath et al., 1973; Kurdziel et al., 2017). Intriguingly, oxygen enrichment partially prevented chronic HAH-induced increase in pulmonary artery pressure and RV systolic pressure, revealing potential protection against pulmonary arterial hypertension. Furthermore, HAH-induced RV remodeling in rats was partially inhibited by oxygen enrichment, characterized by decreased RV weight and reduced cardiomyocyte size. Previous studies only reveal the suppression of heart rate and diastolic arterial pressure of people at high altitudes following oxygen enrichment exposure (Shen et al., 2013; Li and Liu, 2014; Moraga et al., 2018). Our findings further demonstrate that oxygen enrichment has the capability of mitigating the detrimental effects of long-term HAH on cardiac morphology and contractile function in rats.

Substantial studies have confirmed the critical role of hypoxia-inducible factors (HIFs) as key regulators responding to oxygen deficiency (Schofield and Ratcliffe, 2004; Majmundar et al., 2010). In the present study, the mRNA and protein expression of HIF-1*α* (an oxygen-sensitive subunit) in both the LV and RV was significantly increased after chronic HAH exposure. The mRNA levels of Col1α1 and Col3α1 and hydroxyproline concentration in the RV were also greatly upregulated under chronic HAH, whereas no similar change occurred in the LV, revealing that hypoxia increased collagen content and promoted myocardial fibrosis in the RV (Watson et al., 2014). Moreover, the expression of ACE and ACE2 in the LV was both upregulated by HAH, and this balance between ACE and ACE2 (the ACE/ACE2 ratio) maintained the Ang II content (a potent vasoconstrictor). However, the disturbance of the physiological balance between ACE and ACE2 in the RV induced by HAH consequently increased the level of Ang II, which may result in the RV myocyte hypertrophy and cardiac fibrosis (Kim et al., 1995; Schnee and Hsueh, 2000). Oxygen enrichment efficiently mitigated HAH-induced overexpression of HIF-1α in the LV and RV, and also decreased the Col1α1 and Col3α1 expression and cardiac hydroxyproline content in the RV. Furthermore, oxygen enrichment decreased the ACE expression and increased the ACE2 expression in the LV, and thus resulted in the decreased ACE/ACE2 ratio and subsequent decline in Ang II. Although no alteration in ACE2 expression in the RV was observed, the ACE expression and Ang II concentration were decreased following oxygen enrichment exposure. Thus, our findings reveal that oxygen enrichment has the capacity of protecting the LV myocardium from potential damage and also ameliorating the myocardial fibrosis and remodeling in the RV in response to long-term hypobaric hypoxia in rats. Considering the key role of HIFs in oxygen sensing as intracellular transcriptional regulators, we believe that HIFs might act as a metabolic switch mediating the adaptation of cardiomyocytes to oxygen changes, and thus regulate subsequent ACE/ACE2-modulated myocardial remodeling and cardiac fibrosis. The exact HIF-mediated mechanism by which oxygen enrichment improves cardiac function will be identified using gene silencing and overexpressing techniques both *in vitro* and *in vivo* in the succeeding studies.

There are several limitations in this research. During the experiment, it is necessary to change the bedding for the animals and replenish the food and water daily in the hypobaric chamber. Because it is impossible for chamber cleaning and food placement without opening the chamber based on the currently available commercial hypobaric device, short-term exposure (2 h/day in this study) to the normoxic environment for animals seems inevitable. Substantial previous studies have also used the similar experiment protocol with the hypobaric chamber exposed to normoxic environment for daily feeding and cleaning (Pichiule et al., 1996; Titus et al., 2007; Xie et al., 2015). In addition, performing the skin perfusion and behavior open field testing for animals under normoxic conditions is another limitation of this study. Similarly, most of previous animal studies investigating the effects of hypobaric hypoxia on the behaviors or blood perfusion were also performed under normoxic conditions due to various technology challenges for direct testing in the hypobaric hypoxia chamber (Takuwa et al., 2013; Kanekar et al., 2015). Moreover, the study was performed by simulating the condition of HAH in the hypobaric hypoxia chamber in the city of Xi’an. It would be interesting to test the effects of oxygen enrichment on HAH-induced cardiorespiratory dysfunction by simulating the heights at different cities in addition to Xi’an in the future studies, which would allow a more transversal extrapolation of the data.

In conclusion, this study demonstrates that oxygen enrichment can effectively inhibit the augmented skin blood flow and improve the locomotor activity in chronic HAH rats. Oxygen enrichment also has the capability of modifying chronic HAH-induced increased production of erythrocytes. Moreover, oxygen enrichment can attenuate HAH-associated hemodynamic changes and right ventricular hypertrophy in rats. Pulmonary remodeling and vasoconstriction were also significantly inhibited following oxygen enrichment. This study provides the first evidence revealing that the oxygen enrichment technique has the capability of resisting chronic HAH-induced cardiopulmonary dysfunction in animals, and offers preliminary rationale for future clinical trials focusing on evaluating whether oxygen enrichment mitigates chronic HAH-induced cardiopulmonary damage for workers and residents on the plateau. The oxygen enrichment technique can not only be designed for individual use, but more importantly can be designed for providing diffusion oxygen supply in local space (e.g., room, tent, and vehicle) to decrease the equivalent altitude and attenuate chronic mountain sickness.

## Data Availability Statement

The original contributions presented in the study are included in the article/supplementary material, further inquiries can be directed to the corresponding authors.

## Ethics Statement

The animal study was reviewed and approved by Institutional Animal Care and Use Committee of the Fourth Military Medical University.

## Author Contributions

DJ, CT, and EL designed the research. XS, XD, JC, and ZY performed the experiments. XS, XD, JC, ZY, KX, DJ, and EL analyzed data. XS, JC, and DJ wrote the paper. All authors have read and approved the manuscript.

### Conflict of Interest

The authors declare that the research was conducted in the absence of any commercial or financial relationships that could be construed as a potential conflict of interest.
